# Knowledge and technology: A turbulent marriage!

**DOI:** 10.4103/0970-0358.53000

**Published:** 2009

**Authors:** Surajit Bhattacharya

**Affiliations:** Editor, Indian Journal of Plastic Surgery, Senior Consultant Plastic Surgeon, Sahara Hospital, Lucknow, India. E-mail: surajitbh@yahoo.co.in

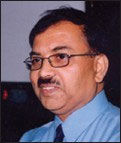



Taking over the mantle of the Indian Journal of Plastic Surgery from my able predecessor and beacon of inspiration, Prof. Mukund Thatte, is indeed an honour and most certainly a difficult responsibility. Not only are we editing an English language Plastic Surgery Journal but we are also inculcating the habit of scientific documentation amongst a new breed of outstandingly talented young Plastic Surgeons, both in India and overseas. Towards meeting these twin objectives, we remain indebted to our meticulous and ever-approachable group of national and international reviewers who have, from time to time, by their pointed questions and timely interjections metamorphosed mundane texts into articles of academic excellence. Thank you all. The new editorial team is starting with a brand new Journal cover, in which it wishes to immortalize the Plastic Surgery icons of yesteryears of our great country. And who else could be the first in the line other than Prof. C. Balakrishnan, a fountain of knowledge and a keen researcher of newer technology!

KNOWLEDGE and TECHNOLOGY are two comets that we are trying to ride at the same time. Both are fast and we have no control over them. We are holding on, with great difficulty, to the searing tails of these two Comets, hoping that they will take us to the right place at the end of the ride. Sometimes we pray that we can hold on for the entire ride. Sometimes we find ourselves praying that Almighty God will be merciful and just let us fall off. What we shudder to think is what will happen the next time the two Comets choose to move in opposite directions as they have undoubtedly done in the past.

While it is so true that we can not hold on to the older proven techniques and should continue to strive for excellence at all times, the reality is that all that is new and available is not invariably good and useful. There is a lot of market driven euphoria and baseless jingoism with quite so called new gadgets, hence our reluctance to succumb to them. When confronted with a new machine / technology we should ask whether we are the masters or the slaves of technology. Once we understand that we must be the master, the rest is fairly simple. The ultimate purpose of any technology is to help its master to do his or her job better. Our job is to cure patients, improve the quality of recovery and patient's well being, and reduce the sufferings of those we can not cure. We also must make treatment efficient and cost effective. If a product of the new technological boom does not meet these criteria, we must ask “Why are we doing this?”

State of the art technology is no substitute for state of the art knowledge; in fact it is quite useless and on most occasions downright harmful without it. Technology has not always taken us to the crest of success. The moment we have allowed it to lead our knowledge, with our subconscious connivance, it has led us astray, or more correctly we have allowed technology to lead us astray. Extracorporeal Shock Wave Lithotripsy (E.S.W.L.) has broken millions of stones in the kidney, ureter, and bladder. Thinking it to be a stone breaking machine, a few amongst us tried to crush stones in the gall bladder expecting them to disappear like magic. We conveniently forgot to compare the anatomy and physiology of the two organs - in one clear urine washes down the debris through a straight ureter and in the other thick viscid bile slowly finds its way down across a serpiginous course and through a valve. The result was a disaster of enormous proportions - all because we did not do our homework well. The marriage of knowledge and technology is a very turbulent one as both are highly ambitious and growing rapidly. A postal delay in the delivery of two journal issues leaves one fairly behind in patient care and ignoring seminars and conferences makes one antique.

If necessity is the mother of invention, then strategy should be its father. Technology should not evolve spontaneously. Its evolution should be our responsibility. Where do we go from here? What technologies are on the horizon? How do we open our minds without closing too many doors? Surgeons are looking at various technology that could enormously improve patient care and even put them out of business; technology that can fix things without touching them, and touch things without seeing them. These are weary yet exciting times!

The future very often arrives faster than expected. In 1996, a renowned biologist, Lee Silver of Princeton University, wrote that it is impossible to clone mammals via cell-nucleus transfer. His book had not even reached the bookshops when scientists of the Roslin Institute in Scotland announced that they had succeeded in cloning ‘Dolly’ the sheep. The best way to predict the future is to invent it. Delightfully mesmerizing visions of the future created by research laboratories, think tanks, science fiction authors, other visionaries and clairvoyant sooth-sayers not only form a matrix for the social perception of tomorrow's world but also open up a plethora of opportunities. Such futuristic thoughts are known as memes, which propagate in society like a cultural gene. Mass media dictates the collective expectations of society, hence they can analyze the memes. Cinema can project new technologies as being real even if they are in the developmental stages and society will accept it, at least in the subconscious mind. The most radical ideas from science and fiction may find solutions to problems that we face in real life. On the other hand, consumer expectations are also programmed in this way. Microvascular transplants of severed limbs were seen in comic strips half a century ago; today they are a reality in a general hospital near you!

Human life might also become programmable. Families will be designed and children selected according to catalogues. The gender of children might be reversible during the course of pregnancy and the little brother may be a robot! Thus, our notion of family happiness might be programmed and human beings would like to make the entire world a theme park where spectacular experience boosters are available. One-minute holidays and artificial hibernation of unproductive times will become the order of the day.

In the healthcare sector of the future, less emphasis will be placed on curing illnesses than on prevention and well-being. Beings and machines will merge; body and consciousness will be rewired. The new combination of natural and artificial hardware and software will create mechanical humans and human machines. Death might become optional with artificial parts increasingly replacing diseased organs. The secret of self-healing will be decoded, changing the treatment pattern of today. But human evolution will continue and result in new intelligent beings.

The future belongs to those who tell the best stories about the future; in other words, only creative thinkers will get an opportunity to contribute towards future professional needs. Plastic Surgery is generally considered to be a skill-based specialty but we have begun to establish our knowledge base. As a knowledge-based specialty, adoption of new technology is a natural extension of our commitment to patient care and not to skill-based bravado. Many new technologies are adopted from other fields *viz*. nanotechnology, lasers, etc. With a solid knowledge base, we will not be afraid of technological failures and will adopt technology as early as possible. Many technologies are popularized by corporate investments, which may not be inherently interested in patients but be more of business opportunities. This profession has to manage the pressures of the market without ignoring the science because ignoring them totally will be fatal to our future growth. Knowledge has to remain a step ahead of technology and riding these two comets, though not easy, has to be done throughout the active professional life of a Plastic Surgeon!

